# Supplementary material for: OsPUB9 Gene Edited by CRISPR/Cas9 Enhanced Resistance to Bacterial Leaf Blight in Rice (Oryza sativa L.)
Source: Int J Mol Sci. 2024 Jun 28;25(13):7145. doi: 10.3390/ijms25137145 (PMC11241066; doi:10.3390/ijms25137145)
Supplement: Supplementary file 1 [file ijms-25-07145-s001.zip › ijms-3004588-supplementary.pdf]

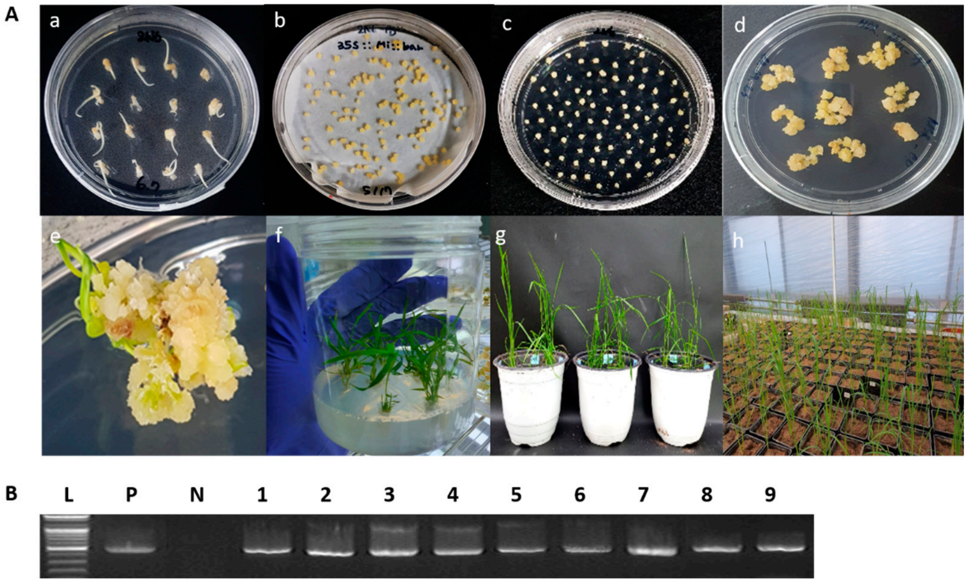

Supplementary Figure S1. (A) Development of transgenic rice plants with CRISPR/Cas9-*OsPUB9* vector using *Agrobacterium*-mediated transformation. (a) Seeds plated on 2N6 media, (b) Callus formation, (c, d) Agrobacterium co-culture in 2N6 AS media, (e) multi-shoot differentiation, (f) Regenerated plants in rooting medium, (g) Acclimation in tissue culture room. (h) Transplanting in greenhouse. (B) Detection of sgRNA/Cas9-mediated DNA modifications using PCR analysis of *bar* and *nos* terminator regions in T_0 generation (GE.PUB9). L, DNA ladder; P, positive control (plasmid vector); N, negative control (sterile water).

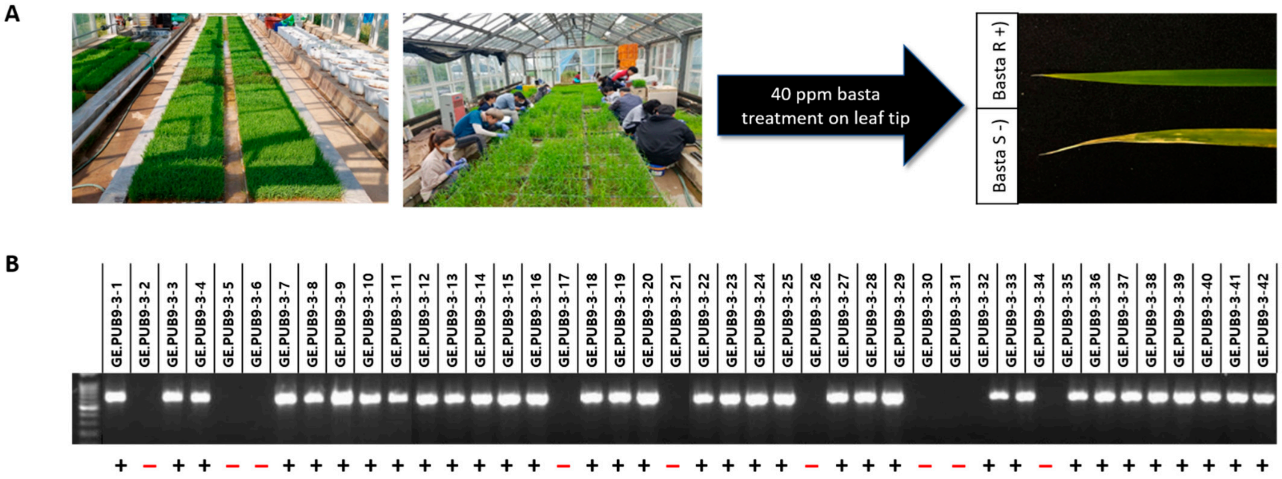

Supplementary Figure S2. Selection of T-DNA-free null lines. *Bar* screening after glufosinate/basta treatment on leaf tip, and *bar* strip analysis in T_1 generation. (A) Screening of *OsPUB9* transgenic T_1 mutants after treatment with 40 ppm basta as followed by Kim et al.(2023). (B) Detection of the selectable marker gene using *bar* PCR. Selection of *OsPUB9* transgenic T_1 plants after *bar* PCR. The minus (-) and plus (+) signs indicate resistance to *bar*.

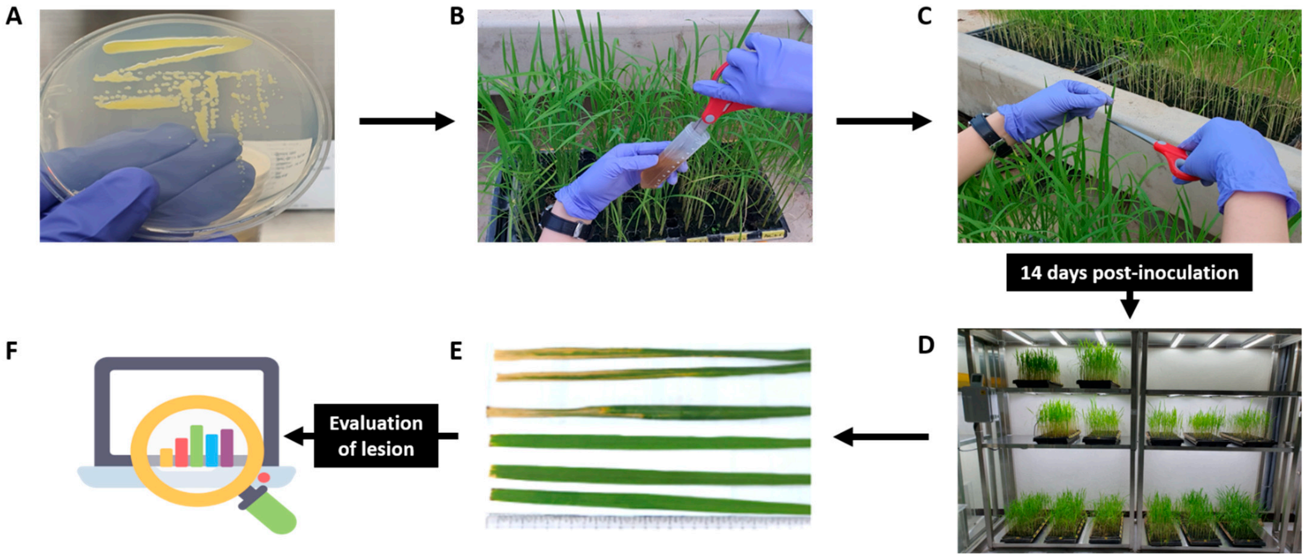

Supplementary Figure S3. Resistance screening of *OsPUB9* gene-edited null lines against *Xoo* K2 strain infection. (A) inoculum made from a 2-day cultured *Xoo* strain K2 on PSA medium, (B) & (C) bacterial suspension used to infect rice leaves using leaf clipping method, (D) phenotypic observation for 14 days post-inoculation in growth chamber, (E) & (F) measurement and evaluation of bacterial blight infected lesion length.

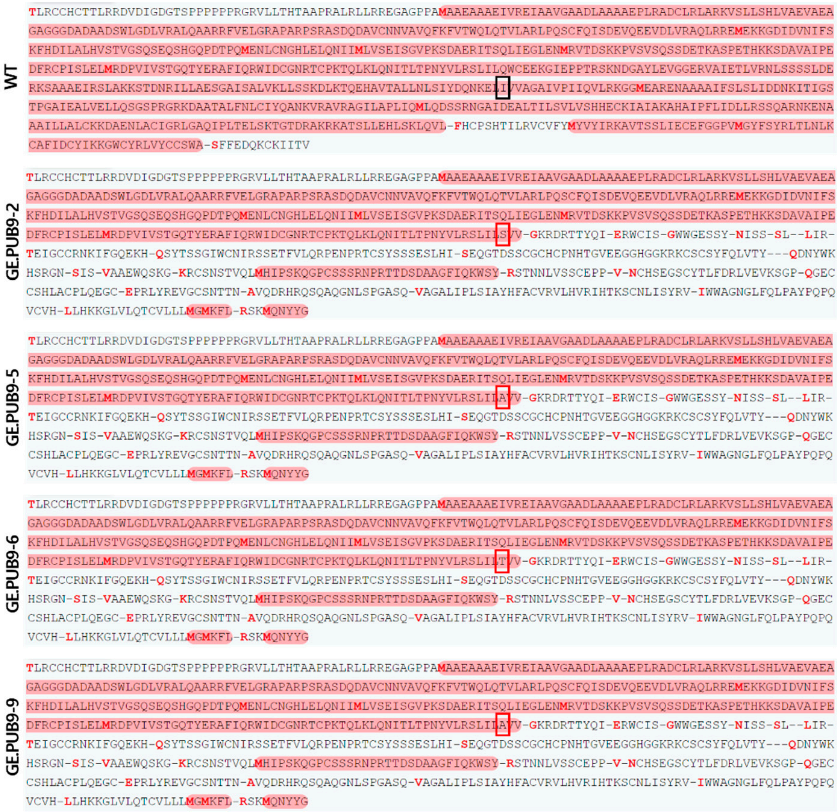

Supplementary Figure S4. Predicted amino acid sequence based on nucleotide sequence of OsPUB9 gene-edited null line (PUB9-2, PUB9-5, PUB9-6, and PUB9-9).

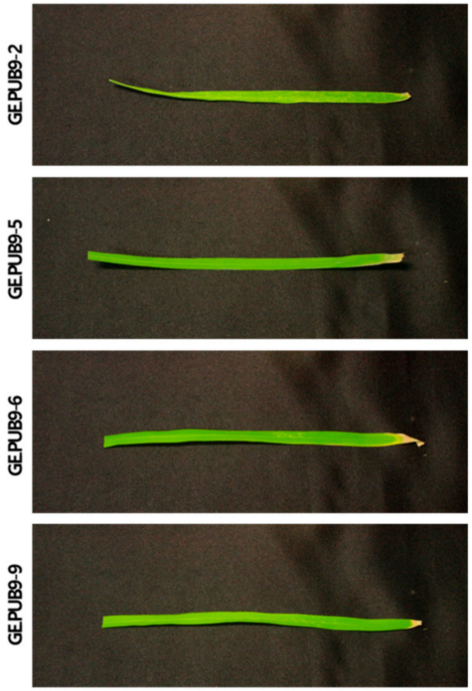

Supplementary Figure S5. Phenotype of bacteria blight (race K2) inoculation in T_2 OsPUB9 gene-edited null lines (PUB9-2, PUB9-5, PUB9-6, and PUB9-9).

Supplementary Table S1. Analysis of *OsPUB9* gene transformation efficiency and mutation rate.

Target gene	No. of transgenic plants	No. of editing plants	Mutation rate (%)[§]	Homozygous		Bi-allelic		sgRNA Seq.
				No. of mutants	Mutant genotype ratio (%)[¶]	No. mutants	Mutant genotype ratio (%)	
OsPUB9	9	5	55.6	1	20.0	4	80.0	tgcgaagtttgatattgcagtgg

[§]Mutation rate (%) = (No. of editing plants/No. of transgenic plants) *100, [¶]Mutant genotype ratio (%) = (No. of editing plants/No. of mutant by genotype) *100.

Supplementary Table S2. Chi-square (χ^2) analysis of the genotypes with *bar* screening from *OsPUB9* T_1 plants.

Rice line	Genotype	No. of resistant plants	No. of susceptible plants	No. of all edited plants	χ^2	p
GE.PUB9-3	Homo	33	9	42	0.29	< 0.5

Supplementary Table S3. Standard evaluation system (SES) scale for scoring data of bacterial blight disease (IRRI, 1996).

Scale	SES (Rating)	Effected lesion area (%)
1	Highly resistnat (HR)	1 - 5
3	Resistant (R)	6 - 12
5	Moderately resistant (MR)	13 - 25
7	Susceptible (S)	26 - 50
9	Highly susceptible (HS)	51 - 100

Supplementary Table S4. The primers list used in this study.

Oligo name	Forward sequence (5′-3′)	Reverse sequence (5′-3′)
XB3	GCGGGAACTTGGATTGCATC	CAAGCAGACCTTGTTTGGGC
XB25	GTGTTCACCCAGATGGCAGA	CACCGTAACCACACGCAAAA
XB21	CGACGCCATCTTTGACTCCT	CCCTTGAGATTTTGCAGGCC
XB24	GCCCTCTGAACTGGTGGAAT	ACCTTCCTCAATGTGCCTGC
xa13	AGTCGACGGGAGGGTACAG	GACGAGGTAGAGGGTGGTGA
xa41	CCTTCGCCTTTGGTCTCCTA	CGCTGATGGTGAGGGAGAAG
Xa10	GCCGTCCATATTGCGATTGT	GGGGAAGAAGAAGGCGAAGA
Xa23	TGCATCATCTCAAGGAGCTGG	AGGGAGAATAACCATCTTGTCGT
Xa27	CGATGCACCACTACCTCCTAC	ACATGTAGGGAGAATGCAAGGA
Xa1	TCGTCACAGGAAGAGGCAAC	ACCGTGTCTTTCAACTTGC
Xa5	GCTCGCCATTCAAGTTCT	GCCACAATCTTCACCTTGCC
NPR1	GCAGGTGAGAGTCTACGAGG	CCGACCTGTCATTCTCCTCC
PUB22	GTCTCGATCCCGACACCATC	TACAGCGTCATCGTCATCCG
XB3	GCGGGAACTTGGATTGCATC	CAAGCAGACCTTGTTTGGGC
XB25	GTGTTCACCCAGATGGCAGA	CACCGTAACCACACGCAAAA
XB21	CGACGCCATCTTTGACTCCT	CCCTTGAGATTTTGCAGGCC
